# Diabetes—Tuberculosis Care in Eswatini: A Qualitative Study of Opportunities and Recommendations for Effective Services Integration

**DOI:** 10.3389/ijph.2023.1605551

**Published:** 2023-03-30

**Authors:** Victor Williams, Alinda G. Vos-Seda, Samson Haumba, Lindiwe Mdluli-Dlamini, Marianne Calnan, Diederick E. Grobbee, Kennedy Otwombe, Kerstin Klipstein-Grobusch

**Affiliations:** ^1^ Julius Global Health, Julius Center for Health Sciences and Primary Care, University Medical Center Utrecht, Utrecht University, Utrecht, Netherlands; ^2^ National Tuberculosis Control Program, Manzini, Eswatini; ^3^ Division of Epidemiology and Biostatistics, School of Public Health, Faculty of Health Sciences, University of the Witwatersrand, Johannesburg, South Africa; ^4^ Faculty of Health Sciences, University of the Witwatersrand, Johannesburg, South Africa; ^5^ Center for Global Health Practice and Impact, Georgetown University Medical Center, Washington, DC, United States; ^6^ University Research Co., LLC, Manila, Philippines; ^7^ Perinatal HIV Research Unit, Faculty of Health Sciences, University of the Witwatersrand, Soweto, South Africa; ^8^ Institute of Tropical Medicine, University of Tübingen, Tübingen, Germany

**Keywords:** non-communicable diseases, tuberculosis, diabetes mellitus, primary healthcare, services integration

## Abstract

**Objective:** This study describes the availability of basic services, equipment, and commodities for integrated DM–TB services, best practices by healthcare workers, and opportunities for better integration of DM–TB care in Eswatini.

**Methods:** A qualitative design was used. Twenty-three healthcare workers participated in a survey and key informant interview.

**Results:** Most respondents indicated DM and TB care are integrated and clients access blood pressure and fasting/random blood glucose assessment. Few respondents indicated they provide visual assessment, hearing assessment, and HbA1c testing. Respondents experienced stockouts of urinalysis strips, antihypertensive drugs, insulin, glucometer strips, and DM drugs in the previous 6 months before the interview. Four main themes emerged from the qualitative interviews—quality and current standards of care, best practices, opportunities, and recommendations to improve integrated services delivery.

**Conclusion:** While DM care is provided for TB patients, the implementation of integrated DM–TB services is suboptimal as the quality and current standards of care vary across health facilities due to different patient-level and health system challenges. Some identified opportunities must be utilized for a successful DM–TB integration.

## Introduction

The World Health Organization (WHO) recommends bidirectional screening for diabetes mellitus (DM) and tuberculosis (TB) (1). In this approach, DM patients are screened for TB while TB patients are screened for DM to identify cases of each condition that could have been missed. Bidirectional screening is vital in low- and middle-income countries (LMIC) with a high TB prevalence and surge in non-communicable diseases (NCD), including DM (2–4). The International Diabetes Federation (IDF) estimates that in 2021, 537 million adults were living with diabetes; 50% of these are undiagnosed, while 75% reside in LMICs (4). This indicates every opportunity for screening should be maximized for improved case finding and treatment as the number of cases is expected to increase to 643 million by 2030 (4). Similarly, TB accounted for 1.5 million deaths in 2020 (5). Before 2020, significant progress was made in the global TB response and countries were on track to eradicate TB by 2035, however, the COVID-19 pandemic reversed this progress (6–8).

DM is a recognised risk factor for TB (9–11). Conversely, TB disease process or its treatment is recognised to alter glucose metabolism resulting in impaired blood glucose (9, 12, 13). Hence, the recommendation for bidirectional screening and integrated management by WHO (1). Different LMICs have integrated bidirectional screening and treatment services for the two conditions with varying outcomes (14–17). WHO defines integrated services delivery as “the management and delivery of health services so that clients receive a continuum of preventive and curative services, according to their needs over time and across different levels of the health system” (18). Services integration offers several benefits. First, in the early identification of cases to limit the spread of infection and development of complications; second, early identification and treatment of DM can reduce the risk for TB infection; third, integration can help optimise the treatment outcomes and retention in care for the different conditions; fourth, integration can improve documentation, monitoring, and reporting of DM and TB, and finally, it can limit resource requirement for health services delivery (19).

In Eswatini, the HIV prevalence is 24.8% in adults aged 15 years and above (20). Consistent with the high HIV prevalence is the TB incidence of 319/100,000 as of 2021 (21). Available data from IDF indicates the prevalence of DM is 3·6% in adults, and the age-adjusted prevalence of impaired glucose tolerance is 6.9% (22). A recent study among outpatient attendees at a tertiary health facility in Eswatini indicates the prevalence of DM and impaired glucose tolerance is 7.3% and 6.5% respectively (23), and 6% and 30% respectively in HIV patients (24). An estimated 15% of outpatient visits in 2020 were for NCDs, and 4% of these were related to DM (25). Data on the prevalence of DM in TB patients is not available.

Before the COVID-19 pandemic, there was a limited effort at integrating DM–TB, but in 2021, tools for the screening of TB patients for NCDs including DM became available. This process is still in its infancy with limited data available to assess the level of implementation or guide practice and policy for TB patient care. This study assesses the integration of DM care into TB services at select health facilities in Eswatini and describes the availability of basic services, equipment, and commodities for integrated TB–DM services delivery, best practices by healthcare workers, key challenges and recommendations on how services can be improved.

## Methods

### Study Design, Setting, and Participants

This qualitative study (with a pilot survey) is part of an ongoing prospective cohort study of newly enrolled clients in TB care in Eswatini from June 2022 and is based on the social-ecological model. The social-ecological model explores various relationships—individual, interpersonal, community, organisational, and policy/environmental factors which determine the health outcome of an individual (26, 27). Healthcare workers from twelve health facilities were purposively selected to participate in the study—a pilot survey followed by a semi-structured interview. The health facilities were selected from the four regions of Eswatini based on the highest average number of new tuberculosis patients enrolled in the two quarters before this study. These consisted of five hospitals, one health centre, and six primary care clinics.

The healthcare workers were invited to participate if they were doctors or nurses, worked in the TB clinic for at least 1 year, and directly provided routine care to TB clients. A minimum of two healthcare workers (one doctor and one nurse) who met the above criteria were purposively selected from the TB clinic for interview per health facility. It was expected saturation would be achieved after interviewing 20–30 participants (28).

### Data Collection and Interviews

Healthcare workers who met the selection criteria and consented to participate were interviewed between May and June 2022. They were requested to respond to a short survey administered through REDCap (29) before the interview. The survey obtained basic demographic details of the healthcare worker, the availability of certain services, and basic requirements for DM-TB care at their health facilities (Supplementary File S1). A semi-structured interview guide was used to obtain healthcare workers’ views on DM–TB care, challenges during service delivery and recommendations for improvement (Supplementary File S2). A trained research assistant fluent in English and Siswati conducted the interviews which were also recorded. Healthcare workers expressed themselves in either English or Siswati. Interviews were conducted face-to-face or telephonically and lasted 30–45 min. The study was approved by the Eswatini Health and Human Research Review Board (EHHRRB-036/2021). Informed consent by participants also covered the interview recording. Identifiable details of participants were not taken to maintain privacy and confidentiality.

### Qualitative Analysis

Recordings from the interviews were transcribed verbatim in English immediately after each interview. The study Principal Investigator (PI) and the research assistant reviewed each interview recording and transcript for accuracy before analysis. The PI documented predefined codes (deductive) and those identified by the participants during the interview (inductive) in a codebook (VW). Analysis of each interview transcript was used to refine the different codes (VW). Four co-authors (SH, MC, AV, and KO) reviewed samples of the transcripts with the identified codes for accuracy and consistency of the identified codes. A consensus resolved any disagreement on the codes. VW and AV independently grouped the codes into themes and all the authors approved the themes once the coding was complete. Themes were categorised and presented using text and summary tables. NVivo 12 software was used for qualitative analysis (30).

## Results

**FIGURE 1:**
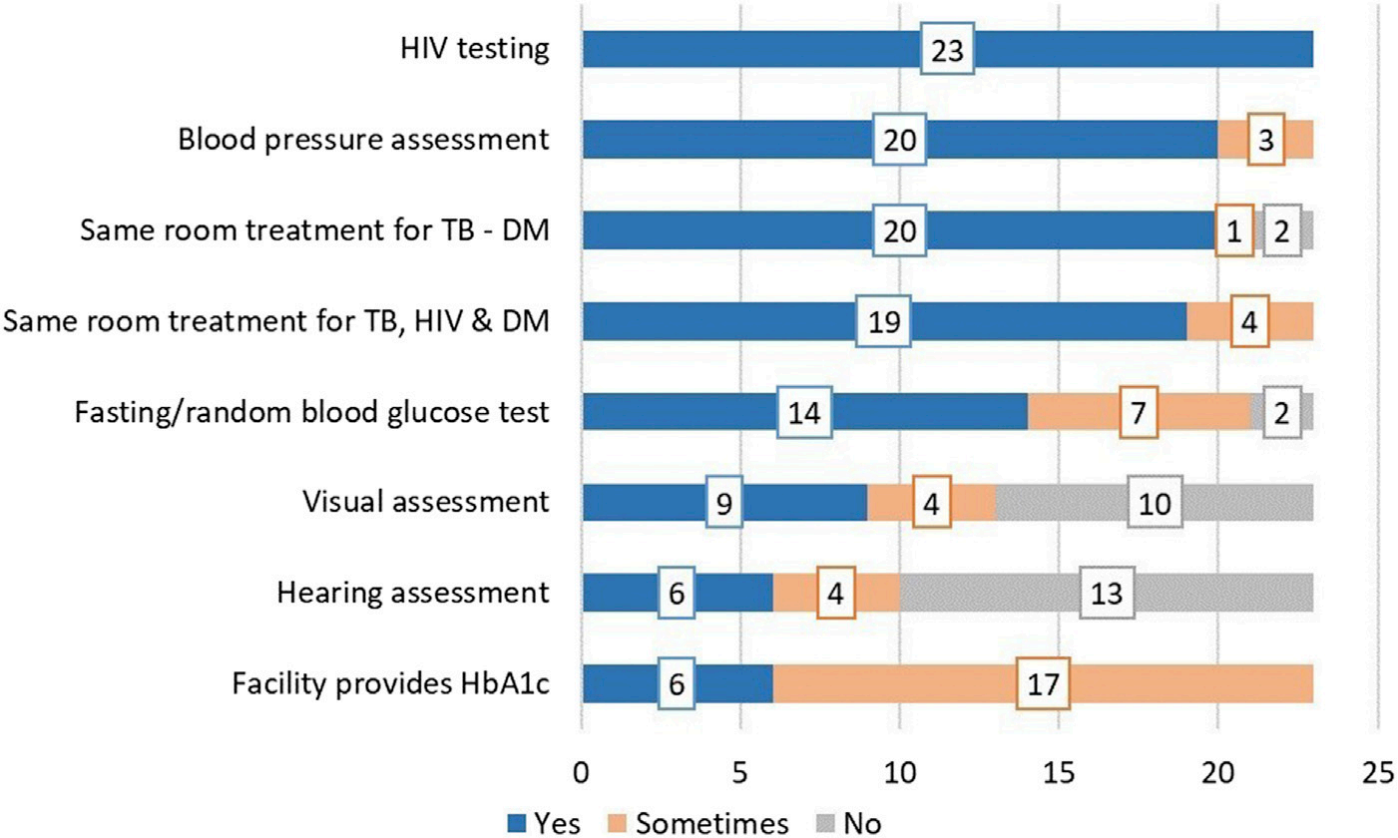
NCD-related services provided at baseline in addition to routine TB services (Eswatini 2022).

**FIGURE 2:**
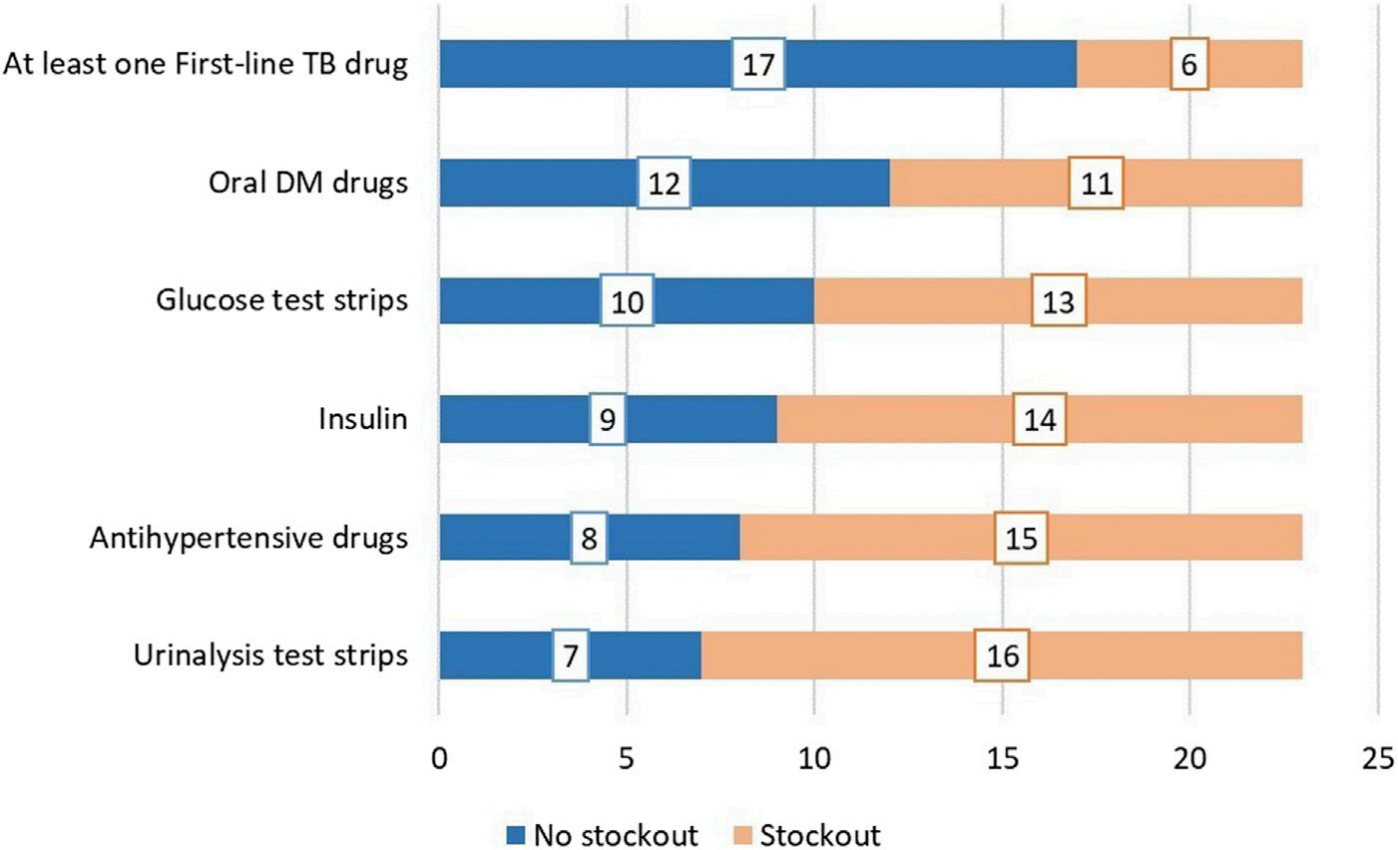
Availability of essential commodities and medication for the management of NCDs within the last 6 months before the interview (Eswatini, 2022).

Of the twenty-five healthcare workers from 11 health facilities invited to participate in the survey, 23 (92%) accepted to be interviewed. One health facility did not respond, and one healthcare worker was unavailable for the interview. Table 1 summarizes the sociodemographic details of the healthcare workers while other findings from the pilot survey of the healthcare workers who were interviewed are summarized in Figures 1, 2.

**TABLE 1:** Demographics and experiences of healthcare workers in the study (Eswatini, 2022).

Variable	Doctors (*n* = 10)	Nurses (*n* = 13)	Overall (*n* = 23)
Age			
Mean (SD)	40.80 (8.4)	36.77 (8.6)	38·52 (8.6)
Min, Max	32.0, 59.0	26.0, 56.0	26.0, 59.0
Gender of healthcare worker			
Female	2 (20.0%)	8 (61.5%)	10 (43.5%)
Male	8 (80.0%)	5 (38.5%)	13 (56.5%)
Highest qualification attained			
Certificate	0 (0.0%)	1 (7.7%)	1 (4.3%)
Degree	4 (40.0%)	6 (46.2%)	10 (43.5%)
Diploma	0 (0.0%)	4 (30.8%)	4 (17.4%)
Postgraduate	6 (60.0%)	2 (15.4%)	8 (34.8%)
Total years of experience in the profession			
Mean (SD)	13.70 (9.3)	13.08 (8.7)	13.35 (8.8)
Min, Max	4.0, 34.0	4.0, 34.0	4.0, 34.0
Number of years providing care for TB patients			
Mean (SD)	6.80 (4.4)	4.00 (2.1)	5.22 (3.5)
Min, Max	1.0, 15.0	1.0, 7.0	1.0, 15.0
Number of in-service training in the last 12 months			
Mean (SD)	3.00 (4.1)	3.46 (3.0)	3.26 (3.5)
Min, Max	0.0, 11.0	0.0, 12.0	0.0, 12.0
Received training for non-communicable diseases?			
No	2 (20.0%)	5 (38.5%)	7 (30.4%)
Yes	8 (80.0%)	8 (61.5%)	16 (69.6%)
Received training in TB-NCD care?			
No	4 (40.0%)	6 (46.2%)	10 (43.5%)
Yes	6 (60.0%)	7 (53.8%)	13 (56.5%)
Received specific training on TB/Diabetes?			
No	6 (60.0%)	9 (69.2%)	15 (65.2%)
Yes	4 (40.0%)	4 (30.8%)	8 (34.8%)

### Facility Description and Availability of Guiding Documents

About half of the respondents (*n* = 12) indicated they worked at a hospital while 11 worked at a primary care clinic. Fourteen indicated their health facility was in an urban area, 5 in a semi-urban, and 3 were in a rural area. All (*n* = 23) indicated they had the current national TB treatment guideline, 11 had a Standard Operating Procedure (SOP) for the care of TB patients with DM, 21 indicated there was a requirement for staff of the TB clinic to be trained on NCDs and 20 stated they had the essential medicines list.

### Baseline Services for TB Patients

Figure 1 describes NCD-related services provided at baseline in addition to the routine services for TB care.

### Availability of Essential Commodities in the Previous Six Months

Respondents provided information on the availability of essential commodities and medication for the screening, treatment, and monitoring of NCDs. Availability was determined by the occurrence of stock-outs within the previous 6 months before the interview date (Figure 2).

### Qualitative

Four broad themes emerged from the data. These were obtained from several sub-themes based on codes from the healthcare workers’ interviews (Table 2).

**TABLE 2:** Themes and sub-themes derived from different codes in the study (Eswatini, 2022).

Themes	Sub-themes
Quality and current standards of care	Treatment guidelines
	Screening for DM
	HbA1c and Oral glucose tolerance test
	Care of TB—DM patients
	Pill burden for DM patients
	COVID-19 affected services delivery
Best practices	Staff rotation
	Synchronized clinic visits
	Health education
	Integrated services
	Fast track services
	Bidirectional screening and screening for COVID-19
Opportunities to improve integrated services delivery	Patient support
	HR and staffing
	Staff training
	Reporting tools
	DM and TB diagnosis
	Availability of drugs
Recommendations	NCD management guidelines
	Healthcare worker training
	Patient documentation tools
	Improve diagnostics
	Improve drugs supply
	Patient support and Health Education

### Quality and Current Standards of Care

#### Treatment Guidelines

More than half of the participants (*n* = 12) indicated Standard Operating Procedures (SOP) or documents to guide the care of TB patients with DM or other non-communicable diseases were not available.

“The guidelines (TB and HIV) are clear and good for me, but we need a clear guideline on the NCDs aspect because for now, much attention is given to TB and ART forgetting about NCDs.” R3

“We do not have a proper protocol (for TB-DM). We just manage the patient based on what we are doing as our routine practice.” R22

#### Screening for DM

The random blood glucose or fasting glucose test was commonly used by healthcare workers to screen and diagnose DM, and 14 respondents indicated they screen patients at baseline.

“We check the fasting or random blood sugar using a glucometer depending on if the patient has eaten or not… If it was random blood sugar, I can ask the patient to come the following day not eaten so we can do a fasting blood sugar and further review” R10

#### HbA1c and Oral Glucose Tolerance Test

Participants were aware of the HbA1c and the oral glucose tolerance tests, but these were either not available or had a long turnaround time. Only 6 respondents stated they screened patients at baseline with HbA1c.

“…In our facility, we do not perform the HbA1c test and again we do not do the oral glucose tolerance test” R1

“…the HbA1c test is available, but we do not have the reagents, so we are not doing it currently” R11

“The HbA1c test is not run here but in Mbabane, we are usually allocated a day wherein they come and collect the sample. So, if a patient comes on Monday, she has to wait for Friday so that the specimen can be collected” R14

#### Care of TB–DM Patients

The participants had different approaches to providing care for TB patients with DM. A common practice was to refer the patient to a hospital or a doctor if the facility was a clinic or the healthcare worker was a nurse. Additional care includes counselling for lifestyle modification and assessment for complications.

“If the patient has already been diagnosed with DM and is on treatment, we continue with the treatment. But if I test a patient and find that he/she has abnormal blood glucose and is not a known DM patient, I refer that patient to the doctor for review” R10

#### Pill Burden for TB Patients With DM

There was a concern associated with adherence due to the pill burden, and the fact DM care was previously not prioritized until the COVID-19 pandemic.

“…diabetes mellitus needs more attention than HIV these days. HIV is well funded, patients are now taking one pill per day, but with DM, patients are taking 3-4 tablets” R8

“If I can be precise, there has been poor sensitization of DM, it only gained much attention during the COVID-19 era… we knew about DM but there was no clear guidance and proper management.” R7

#### COVID-19 Affected Services Delivery

The participants also described how the different health measures adopted during the COVID-19 pandemic affected healthcare services resulting in sub-optimal patient care.

“If you were living with TB, hypertension or DM during the COVID era, follow-up was poor, because there was an instance where we lowered the volume of people visiting the facility for infection control purposes, hence we were giving patients 3 months, thus in the 3 months no one was following if you have been doing well or if your blood sugar was controlled, so by the end of the 3 months most patients were elevated.” R8

“Patients were not coming to the clinic for monitoring and check-ups due to fear of contracting COVID, this led to other patients being severely attacked by TB and others had their DM escalated” R7

### Best Practices

Participants described some of the best practices they adopted to continue providing healthcare services (Table 3). These include staff rotation, a process where they are routinely reassigned to work in different units and departments within the health facility; synchronizing patients’ clinic visits so they can receive care for multiple health conditions on the same day; routinely providing health education for their patients; and integrating services where they provide multiple services for patients at one service point, so patients do not queue at other service points.

**TABLE 3:** Sub-themes describing best practices by healthcare workers with illustrative quotes (Eswatini, 2022).

Sub-themes	Illustrative quote
Staff rotation	“To ensure continual service provision we do staff rotation, if a TB nurse is not available, one is always assigned to the TB department” R4
	“We do have enough clinical staff because we rotate the nursing staff working on the chronic care site, we provide training to our staff so that they can familiarize themselves on HIV and TB care, this is the same staff that we normally use for the rotation” R7
Synchronized clinic visits	“We allow the patients to start at the DM clinic and then come to the TB clinic so that we can assign the very same date as that of DM refill, this is a way of avoiding an instance whereby the patient has to come for TB/DM treatment at different dates, hence we are treating TB and DM as one package together with ART” R12
Health education	“Health education is important to both the patient and the nurse. On the patient aspect, we need to teach the patient about the importance of insulin treatment for the good control of blood sugar and a good response to the TB drug …we are having a challenge of adherence when it comes to the patients that is why the health education is important” R5
	“We then discuss with the patient what is expected and see if we can start on oral medication, we also articulate on the diet issue.” R14
Integrated services	“ we have integrated the services, such that an ART patient, who has DM and TB is going to get all the services at our department even his/her medication. There is no need of going to the pharmacy or laboratory, we do everything here at our department” R3
	“We have also integrated all the services at one consultation room such that ART, DM, TB and hypertension patients or any NCD patient receive all the services at one point.” R18
Fast-track services	“We do NCDs screening and highlight if that patient has one and then fast-track the patient, this helps us to easily check and manage the patient monthly to avoid complications” R7
	“…we normally fast-track them because we do not want them to queue, everything is provided at one place, and we do not want them to go here and there for their services.” R20
	“But one challenge that we normally face is that patients are not honest when giving such information, they tend to say they have no history or family history of DM, such that when a patient has no family history of, we do not fast-track that patient” R7
Bidirectional TB–DM screening and COVID-19	“…now we have adopted at least to screen all TB patients for DM and so far, we have started picking some that we were missing all along. So, in our diabetic clinic we were not screening our DM patients for TB but now we have started screening all our DM patients when they come for a refill, and we are picking patients who are TB positive, but we have been missing them all along” R11
	“COVID-19 was severe for DM patients, thus in our facility we are now screening every patient above 40 for DM if they are found to be positive, we fast track them to the focal person for NCDs” R20

### Opportunities to Improve Integrated Services Delivery

#### Patient Support

The study participants identified some patient-related factors which may result in poor outcomes. These include non-adherence to medication, late presentation at a health facility for review and diagnosis, denial, limited knowledge and understanding of the disease process, and poor economic status.

“Some of the patients do not accept the diagnoses, hence it becomes difficult to assist the patient who has not accepted his/her condition” R17

“…coming to the dietary control, patients do not have money to buy a healthy diet, thus this has an impact on the blood sugar, because when you are not eating a proper diet the blood sugar goes up” R21

#### HR and Staffing

Most participants indicated inadequate clinical staff and would require additional staff to ease the workload and ensure quality care. They indicated this led to prolonged waiting times for patients who visit the TB clinic for different services.

“…we do not have enough clinical staff, it is only one nurse and one doctor wherein we have to take care of ART, hypertension, DM, and TB… hence we appoint patients to different dates in an attempt to control the patient flow” R18

#### Staff Training

While most of the participants (*n* = 13) stated they have received training for TB–NCD, few (*n* = 8) indicated they have specifically been trained to provide care for DM–TB patients.

“With TB I am well equipped, but I am not with diabetes mellitus, hence I require more training and sensitization in that aspect.” R1

“On the part of DM yes, because I am a focal person for D.M., but I cannot say the same for TB For instance if I initiate a patient on TB treatment and the patient complicates, I normally do not know what to do and when to switch the drugs, I do not have enough exposure to TB” R4

#### Reporting Tools

A few participants indicated they did not have the required patient information sheets. They indicated different versions of the patient documentation card were available, requiring varying patient detail and timelines for documentation.

“The standard of care is okay, there are only issues with the white card, one is detailed, and the other is not, now we do not have the detailed white card which I guess it’s the new one, it is very simple and straightforward” R15

#### DM and TB Diagnosis

Most participants agreed they had challenges screening for and diagnosing DM due to recurrent stockout of glucometer test strips, absence of a glucometer, variable access to the HbA1c due to lack of reagents, and an unfavourable schedule for specimen submission. For instance, only 6 and 14 respondents reported providing baseline HbA1c and a fasting/random blood glucose test for patients respectively. In addition, 16 and 13 respondents reported stock out of urinalysis and blood glucose test strips respectively in the previous 6 months. Additional concerns were the absence of GeneXpert cartridges, the long turnaround time for sputum culture, and the limited availability of TB-lam.

“We do face numerous challenges, taking for instance, last month we had challenges with cartridges in the laboratory which made it difficult to transition a patient from an intensive phase to a continuation phase…” R2

“… we do not have a glucometer and a BP cuff such that when a patient comes, you need to run around borrowing from other departments and we end up not doing the proper routine care” R10

“We normally run out of glucose strips.” R16

#### Availability of Drugs

The participants acknowledged frequent stock-out of medication for the treatment of NCDs and tuberculosis. From the survey, the participants indicated a stockout of antihypertensive medication, insulin, and oral DM drugs within 6 months of the date they were interviewed.

“… shortage of insulin; some patients who are taking the 500mg doses (Metformin) are sometimes given the 850mg doses due to shortage of stock” R3

“…for the past 2 months we did not have metformin 500mg which is a drug that we normally give to TB patients who are diabetic, hence this is a rural area patients cannot afford private pharmacies” R17

### Recommendations

Participants provided recommendations on how DM–TB services integration can be improved. This is summarized in Table 4.

**TABLE 4:** Sub-themes describing recommendations by healthcare workers with illustrative quotes (Eswatini, 2022).

Sub-themes	Illustrative quote
NCD management guidelines	“The guidelines (TB and HIV) are clear and good for me, but we need a clear guideline on the NCDs aspect because for now much attention is given to TB and ART forgetting about NCDs” R3
	“I think the national guidelines of TB care are really good, and we refer to them a lot, and they have been updated I think around 2019, but we are struggling on the management of TB and, I feel like if there can be proper guidance to that aspect” R9
	“… there should be a proper guideline on the NCDs aspect” R21
Healthcare worker training	“… I am more equipped in DM; I think more training needs to be done in the area of NCDs since it is where most of us are lacking” R1
	“I would not say I am well equipped on balancing TB and DM, my knowledge is mostly on TB hence I am lacking training on the DM aspect, thus I suggest that more training must be done on the DM aspect since I only know the general aspect of DM such that if I can face a patient who is critical with DM I would not know how to assist.” R2
	“I suggest that there should be more training for healthcare workers, more in-service training is also important as well. I strongly suggest that facilities must be visited by the doctor at least twice a month” R17
Patient documentation tools	“… the MoH needs to ensure that all the required tools (documentation) are available in the facilities as stipulated in the national guideline.” R6
Improve diagnostics	“One can advise that screening TB patients for DM is important but not only using the random or fasting blood sugar but using the oral glucose tolerance test” R5
	“I would also strongly suggest that the HbA1c test be made available to facilities, it is very accurate for diagnosing DM and also to monitor patients who are living with DM” R11
	“… we also need the HbA1c test because for now, we are not doing the best care whether for TB or DM patients” R21
Drugs	“…also availing the medication because it’s one of the many challenges that we see, one drug being available then the next day it’s not then you are supposed to use another drug that will not help” R21
Patient support and Health Education	“I think it (standard of care) can be improved, especially supporting the patients with food since some of them are facing diet issues” R13
	“… health education, regardless of the shortage of drugs we have also noticed that patients are not eating well, and they must be taught about the importance of a balanced diet and that of exercise” R5

## Discussion

Most of our survey respondents (*n* = 20) indicated they have integrated DM care into TB services, but the implementation varies as some services are not routinely available with frequent stock-out of essential medication and commodities required for the care of TB patients with NCD comorbidities (Figures 1, 2). The participants indicated there are no standardized guidelines and SOPs for the management of TB patients with any of the NCDs including DM. The random/fasting blood glucose test was commonly used for screening and diagnosis, while there is limited access to HbA1c required to estimate glucose regulation in the past 3 months and follow-up. Participants were concerned the different limitations they encountered may impact patient outcomes and hinder Eswatini from achieving the goal of ending the TB epidemic.

Some opportunities and recommendations for improvement by the participants include the provision of a uniform guideline and documentation card for the management of TB patients with NCDs, patient support to address adherence and economic burden, additional human resources, training, and capacity building of healthcare workers on the management of TB patients with NCDs, increased access to and availability of TB and DM diagnostics, and improved supply chain management processes to limit the stock out of essential drugs and commodities. The participants also indicated some best practices they adopted to ensure efficient services.

A 2021 study assessing the implementation of recommendations from WHO’s Collaborative Framework for Care and Control of Tuberculosis and Diabetes (14) indicates evidence is available from only 35 countries (out of 194 countries registered with WHO). The authors observed bidirectional screening for the two conditions is possible but that there was limited integration with a parallel care system for the two conditions, absence, and limited knowledge of guidelines for healthcare workers, limited knowledge of DM–TB care amongst healthcare workers, and more emphasis on screening than management. These findings are similar to our research findings, except that, in Eswatini, the services are integrated though the implementation is varied.

An Ethiopian study to explore health system challenges and opportunities for integration of DM–TB care indicates healthcare workers had the motivation to provide integrated DM–TB services but encountered challenges with the continuity of care for DM–TB patients, limited knowledge, and skills in providing care, recurrent stock-outs of supplies for DM care, limited attention to DM, poor data management, and the inability of patients to pay for services (31). Another study from India to explore stakeholder perspectives on challenges and opportunities for integrated DM–TB care indicates that integrated DM–TB care requires improvement in laboratory and diagnostics, drug management, human resources and training of healthcare workers, data infrastructure, and higher-level coordination (32). Findings from our study highlighted the need to also address these gaps.

Patient factors reported in Ethiopia were also reported in studies conducted in Zimbabwe and Pakistan, where transportation costs or long distances to the health facility to access a test and the cost of the test hindered patients from visiting a health facility for a repeat test (33, 34). This is particularly important as TB is more prevalent in people of low socioeconomic status (SES) which has been shown to negatively impact treatment outcomes (35). In our study, the participants expressed concerns as the patient’s economic status limits their access to proper nutrition. This emphasizes the need for continued patient support and the adoption of the patient-centred care model in planning integrated DM–TB services (36).

Studies from Malawi and Angola indicate that integrated DM–TB services can be beneficial (15, 17). In Malawi, they observed no loss to follow-up where services are integrated compared to where there is none or limited integration (14.8%) with higher retention in care of 62.5% after 2 years for people with diabetes where there is integration compared to 41.8% in sites with no integration (17). The study from Angola aimed to assess the burden of NCDs amongst TB patients and pilot the integration of diabetes and hypertension screening within the TB program. They observed a high burden of NCDs amongst TB patients and noted that the absence of screening guidelines and protocols to guide patient management limited the implementation of integrated TB–NCD services (15).

Using a qualitative design enabled the collection of comprehensive information on the DM–TB integration. We selected health facilities and healthcare workers across the four regions to ensure adequate representation. To ensure findings will be useful for TB programming, the study focused on the TB program’s key priorities following engagement with the TB program. Finally, the study team consists of diverse expertise, including qualitative research and TB programming.

Our study has some limitations. First, healthcare workers from non-selected sites (including some private health facilities) may have had different views based on their training and experiences. Secondly, we did not include community-based organizations that mainly see patients on an outreach basis. Healthcare service delivery in community settings is different from facility-based care and presents different opportunities that could have been included for improvement. Finally, we did not include other healthcare workers who support patient care such as pharmacists, laboratory scientists, adherence, and psychosocial officers, etc. These staffs provide vital services along the care continuum and interact with patients. Their views could have added to describing the care processes and some opportunities that could be improved overall. Nevertheless, we believe the evidence provided in this study will be vital to improving DM–TB care in Eswatini and other LMICs.

### Recommendations

The healthcare workers have identified different opportunities but some of these, e.g., additional human resources, and new laboratory equipment are resource intensive and may require long-term planning to achieve. However, some immediate actions can be taken to ensure improved service delivery. First, is developing a standardized treatment algorithm to guide healthcare workers on how to care for TB patients with NCDs including hypertension and DM. This can be adapted from the guidelines provided by TB Union while ensuring the local context is incorporated (37). Healthcare workers can be trained on this standardized algorithm with more experienced clinicians serving as mentors. Secondly, the TB program collaborating with the health promotion unit can develop standardized information, education, and communication (IEC) materials for comprehensive patient education that will include information on NCDs, and nutrition based on the staple food in Eswatini.

Third, indicators for monitoring NCDs amongst TB patients should be tracked in the national reporting system. This will ensure the availability of NCD data for TB patients, and a routine review of this data by the program and stakeholders will identify program needs and guide interventions. Finally, stock out of drugs and other commodities hinders effective care for patients. The procurement process for pharmaceutical commodities can be complex and prolonged. This became worse as the COVID-19 pandemic impacted global supplies. One way of addressing this is to train and mentor healthcare workers to keep accurate records and timely orders of supplies once they have the recommended minimum stock. Issues around financing for the procurement of drugs and laboratory supplies are complex and require multisectoral engagement.

### Conclusion

There is limited implementation of DM–TB integration at health facilities included in this study as the quality and current standard of care varies across health facilities. While challenges and opportunities exist to improve the implementation of DM–TB integration, healthcare workers currently adopt different practices to ensure continued service delivery. Addressing the different patient-level and health system challenges and utilizing the available opportunities is vital for successfully implementing the DM–TB services integration.
