# Corrigendum: Diabetes—Tuberculosis Care in Eswatini: A Qualitative Study of Opportunities and Recommendations for Effective Services Integration

**DOI:** 10.3389/ijph.2024.1606951

**Published:** 2024-01-24

**Authors:** Victor Williams, Alinda G. Vos-Seda, Samson Haumba, Lindiwe Mdluli-Dlamini, Marianne Calnan, Diederick E. Grobbee, Kennedy Otwombe, Kerstin Klipstein-Grobusch

**Affiliations:** ^1^ Julius Global Health, Julius Center for Health Sciences and Primary Care, University Medical Center Utrecht, Utrecht University, Utrecht, Netherlands; ^2^ National Tuberculosis Control Program, Manzini, Eswatini; ^3^ Division of Epidemiology and Biostatistics, School of Public Health, Faculty of Health Sciences, University of the Witwatersrand, Johannesburg, South Africa; ^4^ Faculty of Health Sciences, University of the Witwatersrand, Johannesburg, South Africa; ^5^ Center for Global Health Practice and Impact, Georgetown University Medical Center, Washington, DC, United States; ^6^ University Research Co., LLC, Manila, Philippines; ^7^ Perinatal HIV Research Unit, Faculty of Health Sciences, University of the Witwatersrand, Soweto, South Africa; ^8^ Institute of Tropical Medicine, University of Tübingen, Tübingen, Germany

**Keywords:** non-communicable diseases, tuberculosis, diabetes mellitus, primary healthcare, services integration

There was a mistake in Figure 1 and Figure 2 as published. Figure 2 was used as Figure 1, while Figure 1 was used as Figure 2. The corrected Figure 1 and Figure 2 appear below.

**FIGURE 1:**
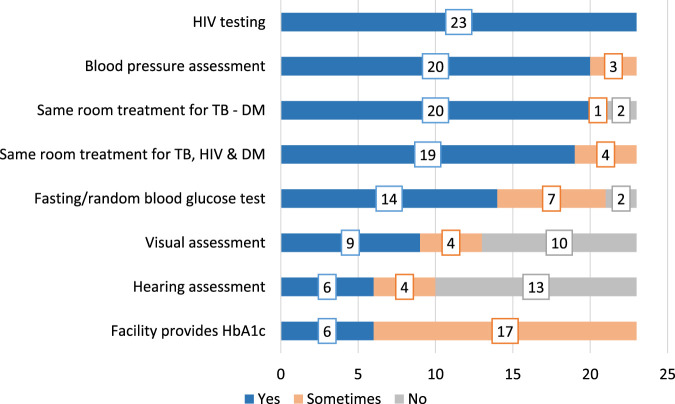
NCD-related services provided at baseline in addition to routine TB services (Eswatini, 2022).

**FIGURE 2:**
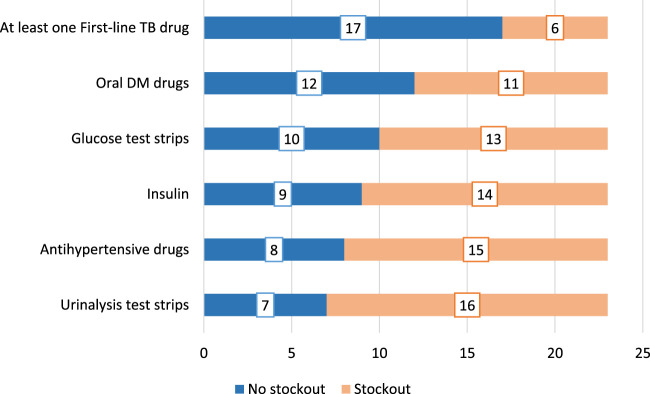
Availability of essential commodities and medication for the management of NCDs within the last 6 months before the interview (Eswatini, 2022).

The authors apologize for this error and state that this does not change the scientific conclusions of the article in any way. The first published incorrect version of the article has been updated.

