# Supplementary material for: Diabetes—Tuberculosis Care in Eswatini: A Qualitative Study of Opportunities and Recommendations for Effective Services Integration
Source: Int J Public Health. 2023 Mar 30;68:1605551. doi: 10.3389/ijph.2023.1605551 (PMC10097913; doi:10.3389/ijph.2023.1605551)
Supplement: Supplementary file 1 [file DataSheet2.PDF]

Healthcare worker's Interview guideline

This guideline provides an outline of the different questions the healthcare worker should be asked based on the different sections of the questionnaire. The researcher should adapt the questions and may not need to necessarily ask exactly the way it is written and should observe the healthcare worker for guiding cues during the interview.

This interview aims to obtain the healthcare worker's perspectives on services accessed by TB clients during treatment. The interview will also identify best practices instituted by the healthcare workers to improve services, challenges they encounter during service delivery and some recommendations on what they think can be done to address the challenges.

Section A: Introduction

The first part of the interview aims to develop a rapport with the participant you are interviewing. It is important to develop a non-judgemental tone throughout the interview and to convey that there are no right or wrong answers.

Start by ensuring the participant is comfortable and at ease. Introduce yourself and confirm you have the right healthcare worker for an interview. Provide a recap about the study and give the healthcare worker a copy of the Study information sheet to review and the consent form to sign if they accept to participate in the study.

Turn the digital recorder on.

Example introduction – adapt as appropriate

Thank you for agreeing to be interviewed for our study today. As I explained earlier, we are studying the different processes involved in the provision of services for TB clients and, those with DM or those who develop DM during treatment. We are interviewing you to better understand this process and some challenges you encounter. This will enable us to develop recommendations that can help improve service delivery in the future.

We are interested in your opinion today; everything you say is very important to us. I will not talk much, but I want you to talk freely, and as much as you want. There are no "good" or "bad" answers.

Section B: Survey to provide a background on the healthcare worker and services delivery

This part of the interview aims to understand the background of the healthcare worker, processes adopted in the care of TB patients with TB, and the availability of optimal work conditions which can enhance services provision.
Open the questionnaire on the tablet and allow the healthcare worker to respond to the short survey questions.

Provide clarity for any question that may not be clear.

DM – TB Care Integration Study ***Participant code: _______***

Section C: Qualitative Interview:

Healthcare provider's perspectives on services delivery

This part of the interview aims to understand how well equipped and confident health practitioners are in providing care for TB clients also receiving treatment for DM. This section will also elicit challenges encountered by health providers, innovative approaches adopted to solve problems and their recommendations for improving service delivery for TB clients and those also receiving treatment for DM. There will also be a further enquiry on the impact of the COVID-19 pandemic on the provision of TB services and how this has impacted service delivery for TB clients with DM.

Now, I would like to ask you some questions about your work, potential challenges, and some recommendations for improvement.

Availability of diagnostics

1. If your patient presents with symptoms of diabetes, what tests are available to help you and your team make a diagnosis?

    Prompt – apart from Fasting blood glucose, do you have an HbA1c test available? If yes, describe the procedure for referring a patient for this test. Is an Oral glucose tolerance test available? Can it be done?

Referral process

2. If a TB patient has diabetes or develops abnormal blood glucose, describe the protocol or referral process adopted for the care of this patient.

Staffing

3. Considering the number of TB Patients seen daily at your TB department, do you think your department has enough clinical staff?

    Prompt- How do you work to ensure continued service provision?

Best practices

4. What best practices have your unit adopted that have improved the care of TB clients receiving treatment in general and those with Diabetes Mellitus?

5. What is your view of the current standard of care for TB patients?

    Prompt – do you think this can be improved? If yes, what can be done?

Training and capacity for services

6. Do you feel you are well trained and prepared to provide the required care for TB clients with DM?

Prompt – if yes, what training have you received? How has it helped you? If not, what training do you specifically need?

Challenges for services delivery

7. Are there any challenges that hinder you from providing effective care to TB Patients in your hospital?

8. Are there any challenges that limit the provision of diabetes mellitus care to TB patients in your hospital?

    Prompts for 7 & 8: What would you suggest that can improve service delivery for TB patients in general and those with diabetes mellitus?

Impact of COVID-19 on services delivery

9. In the last 18 months, how has the COVID-19 Pandemic affected your ability to provide care to TB patients with hypertension and diabetes mellitus?

    Prompt – is there anything that should have been done to improve the care you provide? Which issue stands out for you that should be addressed? What best practice can you recommend to other facilities?

Follow-up of TB patients after the end of treatment

10. Additional comments

Ending the Interview

Before closing the interview, allow the participant to make any further comments about the topics discussed or to ask questions.
Thank the participant for his/her time and for sharing experiences and views.

Switch off the recorder.
