# Supplementary material for: Diabetes—Tuberculosis Care in Eswatini: A Qualitative Study of Opportunities and Recommendations for Effective Services Integration
Source: Int J Public Health. 2023 Mar 30;68:1605551. doi: 10.3389/ijph.2023.1605551 (PMC10097913; doi:10.3389/ijph.2023.1605551)
Supplement: Supplementary file 2 [file DataSheet1.PDF]

Healthcare worker Survey

Record ID ______________________

Date of survey ______________________

Questionnaire code ______________________

Facility type
- ◯ Clinic
- ◯ Health Center
- ◯ Hospital

Facility location
- ◯ Urban
- ◯ Semi-urban
- ◯ Rural

Section B: SURVEY

Sociodemographic details and Occupational History

Age ______________________

Gender
- ◯ Female
- ◯ Male
- ◯ Other

Region of residence
- ◯ Hhohho
- ◯ Lubombo
- ◯ Manzini
- ◯ Shiselweni

Region hospital is located
- ◯ Hhohho
- ◯ Lubombo
- ◯ Manzini
- ◯ Shiselweni

Occupation
- ◯ Nurse
- ◯ Doctor

Nature of work
- ◯ Direct patient care
- ◯ Programmatic
- ◯ Other

Highest education qualification
- ◯ Certificate
- ◯ Diploma
- ◯ Degree
- ◯ Postgraduate

Total number of years in profession ______________________

Number of years providing care for TB patients	__________________
Have you received training for non-communicable diseases?	◯ No<br>◯ Yes
Have you been trained on TB/Non-communicable disease care?	◯ No<br>◯ Yes
Have you been trained specifically for TB/Diabetes?	◯ No<br>◯ Yes
Number of in-service training received in the last 12 months	__________________

Patient care related questions

How many clinical staff (doctors and nurses) provide care for TB patients in your hospital?	__________________
In a month how many TB patients on average present with a non-communicable disease (NCD) in your facility e.g., hypertension, diabetes, asthma	__________________
What is the commonest non-communicable disease they present with?	◯ Asthma<br>◯ Cancer<br>◯ Diabetes<br>◯ Hypertension
In a month how many TB patients present with Diabetes Mellitus (This is baseline blood glucose >7.0 mmol/dl)?	__________________

Indicate Yes/No if the following are readily available at your health facility:

	No	Yes
Policy document on TB care	◯	◯
Current National TB Treatment guidelines	◯	◯
Standard Operating Procedure (SOP) for the care of TB patients with diabetes mellitus	◯	◯
Training requirement for staff on non-communicable diseases	◯	◯
Essential medicines list	◯	◯

If a TB patient has DM, is the treatment offered at the same consultation room?	◯ No<br>◯ Yes
Does your facility provide Hba1c?	◯ No<br>◯ Yes
Does your facility provide fasting/random blood sugar test?	◯ No<br>◯ Yes

Indicate Yes/No if the following services are offered to TB clients at baseline:

	No	Yes
HIV Test		
Fasting/random blood sugar		
Hba1c		
Blood pressure measurement		

Indicate Yes/No if the following services are offered to TB clients during follow-up visits

	No	Yes	Sometimes
HIV Testing			
Fasting/random blood sugar test			
Hba1c			
Blood pressure measurement			

Indicate if the following are available or not available for TB patient screening at your OPD

	Not Available	Available
Sphygmomanometer		
Weighting scale		
Glucometer		
Urinalysis test strips		

Indicate Yes/No if the following medications are available for dispensing at your facility

	No	Yes	Sometimes
Insulin			
Oral Diabetes drugs			
Antihypertensive drugs			
Anti-asthmatic drugs			

Indicate Yes/No if there has been a stock-out of the following medications at your hospital in the last six months

	No	Yes
Glucometer test strips		
Urinalysis test strips		
Insulin		
Oral Diabetes medication		
Antihypertensive medications		
At least one 1st line TB medication		
